# Durable Response to Sotorasib Plus Panitumumab in NRAS G12C-Mutated, Microsatellite Instability-High (MSI-H) Rectal Cancer Refractory to Immunotherapy

**DOI:** 10.7759/cureus.110414

**Published:** 2026-06-07

**Authors:** Latif Karahan, Sevtap Arslan, Omer Dizdar

**Affiliations:** 1 Medical Oncology, Hacettepe University Cancer Institute, Ankara, TUR; 2 Department of Radiology, Hacettepe University Faculty of Medicine, Ankara, TUR

**Keywords:** durable response, immunotherapy, msi-high, nras g12c mutation, rectal cancer, sotorasib and panitumumab

## Abstract

Oncogenic Rat sarcoma (RAS) mutations are common drivers of solid tumors, yet for decades they were regarded as “undruggable.” The development of mutation-specific Kirsten Rat sarcoma viral oncogene homolog (KRAS) G12C inhibitors such as sotorasib has challenged this paradigm and demonstrated clinical activity in KRAS G12C-driven cancers. Neuroblastoma RAS viral oncogene homolog (NRAS) G12C mutations are exceptionally rare in colorectal cancer, and their therapeutic vulnerability remains undefined. Here, we report an objective tumor response to the combination of the KRAS G12C inhibitor sotorasib and epidermal growth factor receptor (EGFR) blockade in a patient with advanced, NRAS G12C-mutated, mismatch repair-deficient rectal adenocarcinoma who developed a local recurrence after neoadjuvant therapy and surgery and subsequently experienced disease progression despite multiple lines of systemic treatment, achieving a durable clinical response lasting more than twelve months. This case provides real-world evidence that NRAS G12C mutations may confer sensitivity to G12C inhibitors combined with EGFR blockade. Molecular testing should include NRAS and Harvey rat sarcoma viral oncogene homolog (HRAS) isoforms.

## Introduction

Oncogenic Rat sarcoma (RAS) mutations, including Kirsten Rat sarcoma viral oncogene homolog (KRAS), neuroblastoma RAS viral oncogene homolog (NRAS), and Harvey Rat sarcoma viral oncogene homolog (HRAS), are among the most common driver alterations in cancer [1]. RAS proteins act as molecular switches cycling between guanosine diphosphate (GDP)-bound inactive and guanosine triphosphate (GTP)-bound active conformations, transmitting proliferative signals through the mitogen-activated protein kinase (MAPK) and phosphoinositide 3-kinase (PI3K) pathways. For decades, these mutations were deemed “undruggable” [1]. The recent development of mutation-specific RAS inhibitors-most notably for KRAS G12C-has challenged this dogma [1]. The G12C substitution impairs intrinsic GTPase activity, locking RAS in a constitutively active state and driving uncontrolled tumor proliferation. Sotorasib (AMG 510) was the first KRAS G12C inhibitor approved by the Food and Drug Administration (FDA) and has demonstrated clear efficacy in KRAS G12C-mutated tumors [1]. In non-small cell lung cancer, compared with chemotherapy, sotorasib significantly improved outcomes in pretreated patients with the KRAS G12C mutation [2,3]. In colorectal cancer (CRC), the KRAS G12C mutation is found in ~3-4% of cases and confers resistance to epidermal growth factor receptor (EGFR)-targeted antibodies [4]. Sotorasib monotherapy has modest activity in KRAS G12C-mutated CRC [5], but combining KRAS-G12C inhibition with EGFR blockade enhances efficacy, has yielded higher response rates in early studies and is now being evaluated in phase III trials [6,7] and recently (in early 2025) approved by the FDA for KRAS G12C-mutated CRC.

In contrast, mutations in NRAS are present in only ~3-5% of CRC cases, and the specific NRAS G12C variant is exceedingly rare (far less common than KRAS G12C) [8]. NRAS mutations (at any codon) have traditionally indicated primary resistance to anti-EGFR therapies in CRC, and until recently, there were no targeted treatments for NRAS oncogenes. Here, we report a case of rectal cancer harboring an NRAS G12C mutation that achieved a clinical response to sotorasib in combination with the EGFR-targeting antibody panitumumab. Written informed consent was obtained from the patient for treatment and publication of this case report in an open-access journal. Ethical approval was not required for this study in accordance with institutional policy.

## Case presentation

A 61-year-old male with no significant past medical history was diagnosed with proximal rectal adenocarcinoma (starting at ~10 cm from the anal verge; cT4N0M0) in March 2023. He had no distant metastasis at the time of diagnosis. He received 4 cycles of neoadjuvant chemotherapy (capecitabine/oxaliplatin) and 5x5 Gy radiotherapy followed by low anterior resection in August 2023. Pathology revealed moderately differentiated adenocarcinoma, ypT1N0, with a near-complete treatment response. Notably, tumor immunohistochemistry revealed the loss of MSH2 and MSH6 expression with intact MLH1 and PMS2, consistent with mismatch repair deficiency and microsatellite instability-high (MSI-H) status. Given the small residual tumor and MSI-H status, adjuvant chemotherapy was omitted. The patient was disease-free until February 2024, when he presented with pelvic pain, purulent rectal discharge, and intermittent fever due to an infected locally recurrent mass. Magnetic resonance imaging (MRI) revealed presacral recurrence, which was not amenable to surgery due to invasion of the sacrum and coccyx (Figure 1).

**Figure 1:**
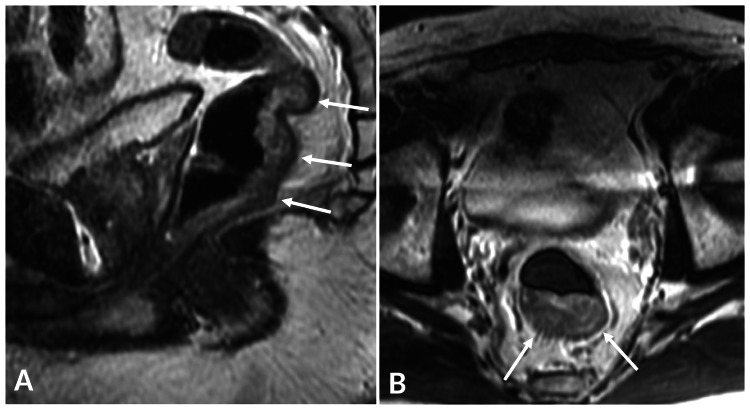
T2-weighted MRI images of the patient (A) Sagittal and (B) axial MR images show a mass (white arrows) in the mid-to-distal rectum.

Given the presence of an MSI-H tumor, the patient was enrolled in a clinical trial of immunotherapy. Pembrolizumab (anti-programmed cell death protein 1 (PD-1)) was initiated in combination with the experimental cytotoxic T-lymphocyte-associated antigen 4 (CTLA-4) inhibitor MK-1308A as systemic therapy. After 2 cycles, pelvic MRI revealed an enlarging necrotic rectal mass and bilateral hydronephrosis. Palliative chemotherapy with FOLFIRI (irinotecan and fluorouracil (5-FU)) plus bevacizumab was given for 4 cycles, which resulted in stable disease. The bulky rectal tumor remained symptomatic and chronically infected, necessitating diversion (colostomy) and antibiotics.

At this time, comprehensive genomic profiling of the tumor was performed. Next-generation sequencing revealed an NRAS G12C missense mutation (detailed genomic findings are summarized in Table 1). Given emerging preclinical and clinical evidence supporting sotorasib's activity against the NRAS G12C mutation [8,9], and in the absence of further standard treatment options, sotorasib 960 mg daily was initiated in February 2025 combined with panitumumab (6 mg/kg intravenously every two weeks), a monoclonal antibody targeting EGFR and infusional 5-FU (2400 mg/m^2^ over 48 hours, administered every two weeks). The patient’s clinical course improved dramatically. Within one month, his rectal pain subsided, and the malodorous rectal discharge diminished, becoming clear and serous. He experienced weight gain of ~5 kg and defervescence from infection. By April 2025 (after ~8 weeks of therapy), follow-up MRI demonstrated significant tumor regression: the size of the pelvic mass had visibly decreased (Figure 2). No new lesions appeared. The only adverse effect was a mild acneiform rash resulting from EGFR inhibition. At the end of the 14-month follow-up, the patient continued on sotorasib, panitumumab, and 5-FU, with an ongoing partial response and improved quality of life.

**Table 1:** Pathology report of the patient Genomic alterations were detected in pathology results. Chr: Chromosome; c.: coding DNA sequence; p.: protein change; Gly: glycine; Cys: cysteine; Arg: arginine; *: stop codon; NRAS: neuroblastoma RAS viral oncogene homolog; FBXW7: F-box and WD repeat domain-containing 7.

Gene	Genomic Location	cDNA	Protein	Functional Effect	Variant Allele Frequency (%)
NRAS	Chr 1, Exon 2	c.34G>T	p.Gly12Cys	Activating	20.0
FBXW7	Chr 4, Exon 8	c.1099C>T	p.Arg367*	Loss-of-function	17.0

**Figure 2:**
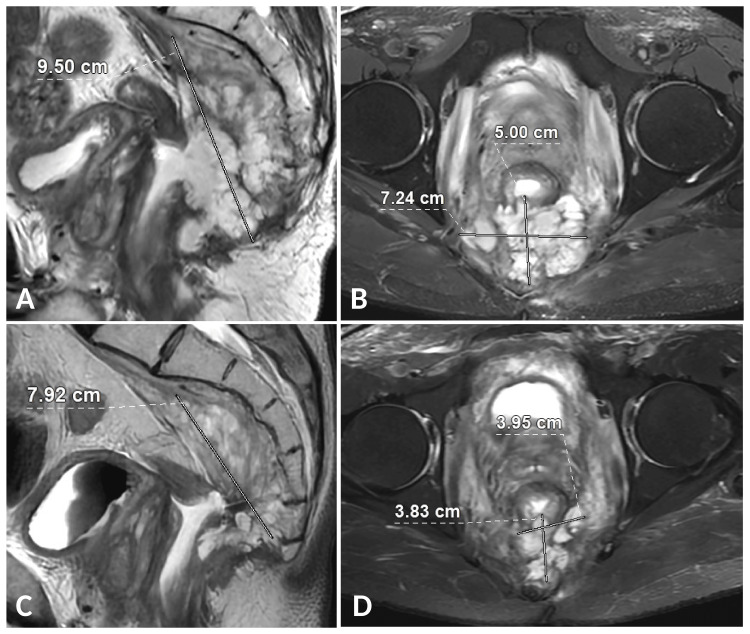
Radiologic response to treatment with sotorasib and panitumumab Pre-treatment (A, B) and post-treatment (C, D) sagittal T2-weighted and axial T2-weighted fat-saturated MR images demonstrate significant tumor regression. The pelvic wall edema secondary to infection has also regressed.

## Discussion

RAS mutations in colorectal cancer are key oncogenic drivers and are present in approximately 40% of cases, with KRAS G12C occurring in ~3-4 % yet associated with poorer outcomes [4]. Recent clinical evidence reinforces the view that, despite its low prevalence, KRAS G12C represents a critical subset of CRC given the emergence of targeted therapies. Importantly, the new KRAS G12C inhibitors raised the question of “cross-reactivity” against other RAS isoforms harboring a glycine 12 to cysteine mutation. The amino acid sequences of KRAS, NRAS, and HRAS are ~85% identical [1], differing in a few residues that might affect inhibitor binding. Preclinical studies have indeed shown that some KRAS G12C inhibitors can target NRAS G12C or HRAS G12C, whereas others cannot [8,9]. Notably, sotorasib binds in a unique pocket (switch-II groove involving histidine 95 of KRAS), and this mechanism appears to translate into pan-RAS activity for G12C mutations [1]. Sotorasib was found to be ~5-fold more potent against NRAS G12C than against KRAS G12C in vitro, likely because NRAS has a leucine at the homologous 95 position, which favors sotorasib binding [8]. By contrast, the KRAS G12C inhibitor adagrasib is highly selective for KRAS and loses activity on NRAS G12C [8]. These findings underscore that inhibitor choice is crucial when targeting uncommon RAS variants.

Until 2023, there were no clinical reports of patients with NRAS G12C-mutated cancers benefiting from KRAS G12C inhibitors [1]. Our case demonstrates, in a real-world setting, that an NRAS G12C-driven tumor can respond to sotorasib-based therapy. Similarly, Möhrmann et al. recently described the first published case of a patient with NRAS G12C mutated locally advanced rectal cancer achieving a clear clinical response on sotorasib plus panitumumab [1]. In that report, a 70-year-old male CRC patient (previously refractory to standard therapies) was treated with sotorasib 960 mg daily and panitumumab, resulting in stable disease at 4 weeks and a confirmed partial response by 12 weeks [1]. This patient’s disease eventually progressed after ~5 months of therapy, but the case provided critical proof of concept that sotorasib can inhibit NRAS G12C in vivo [1]. Our patient’s remarkable improvement corroborates that finding. A recent translational study by Rubinson et al. documented a marked tumor regression in an NRAS G12C colorectal cancer patient treated with sotorasib plus panitumumab, alongside the laboratory data supporting sotorasib’s activity against NRAS G12C [8]. These convergent findings from laboratory and clinical observations highlight the actionable nature of NRAS G12C. To our knowledge, aside from these three cases, there are no other published clinical cases of NRAS G12C-positive cancers treated with G12C inhibitors. However, the NRAS G12C mutation clearly emerges as a potential therapeutic target despite the paucity of evidence.

From a clinical perspective, the patient’s tumor was MSI-H, typically predictive of benefit from PD-1 blockade, yet standard immunotherapy was unsuccessful. After two cycles, rapid disease progression with new-onset hydronephrosis was more consistent with true resistance to immunotherapy rather than pseudoprogression, prompting a change in treatment. Significant clinical and radiological response after targeting the NRAS G12C mutation with a combination of sotorasib, panitumumab, and 5-FU in this case suggests that this combination may be a treatment option for MSI-H, NRAS-mutant CRC.

In summary, this case adds to the literature that NRAS G12C mutations may also respond to sotorasib and panitumumab treatment. Although evidence is currently limited to preclinical studies and a small number of isolated cases, these observations suggest potential therapeutic implications and justify off-label use in refractory situations [1]. Whether other KRAS G12C inhibitors are also effective against NRAS G12C mutations, or whether HRAS G12C mutations can be targeted with KRAS G12C inhibitors, remains an area of investigation [8]. Further research and prospective studies are warranted to establish the efficacy of targeting NRAS G12C with KRAS G12C inhibitors. This case report was prepared in accordance with the CARE reporting guideline, and the CARE checklist was used to guide manuscript preparation and revision [10].

## Conclusions

In conclusion, this case suggests that NRAS G12C-mutated CRC may respond to combined sotorasib and panitumumab therapy, even after immunotherapy failure. This observation highlights a potential therapeutic option in a rare molecular subgroup. Further studies are needed to validate these findings and clarify the role of KRAS G12C inhibitors in NRAS G12C mutations.
